# Supplementary material for: Room-temperature spontaneous superradiance from single diamond nanocrystals
Source: Nat Commun. 2017 Oct 31;8:1205. doi: 10.1038/s41467-017-01397-4 (PMC5663960; doi:10.1038/s41467-017-01397-4)
Supplement: Supplementary file 1 — Supplementary Information [file 41467_2017_1397_MOESM1_ESM.pdf]

Supplementary Figure 1

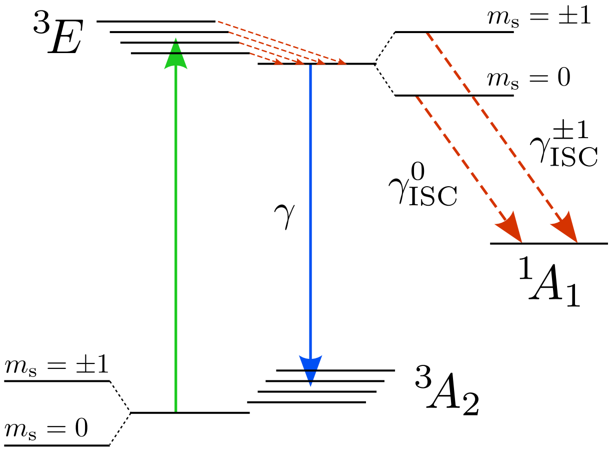

Supplementary Figure 1: **NV-centre energy-level scheme used in the theoretical model**. Solid lines depict optical transitions and dashed lines show phononic transitions. Population in the ground state manifold ($^3A_2$) is off-resonantly excited (green arrow) to a variety of excited states, which then relax down to the excited ^3E manifold over a few tens of picoseconds. Initially this excited population consists entirely of domains that act collectively, but over time local dephasing can move portions of the collective populations into excited, but non-collective states. Population in the ^3E manifold undergoes collective and non-collective optical decay to various phononic sidebands of the electronic ground state ($^3A_2$). The excited manifold can also relax phononically to the $^1A_1$ manifold via the inter-system crossing. As the experimental detection system filters out the ZPL, only photons arising from decay to the excited phononic sidebands of the electronic ground state are seen. Excitation and decays preserve the spin projection quantum number $m_s \equiv \sigma$, so population in the $m_s = 0$ and $m_s = \pm 1$ levels are treated independently and their fluorescence added to produce the final result.

Supplementary Figure 2

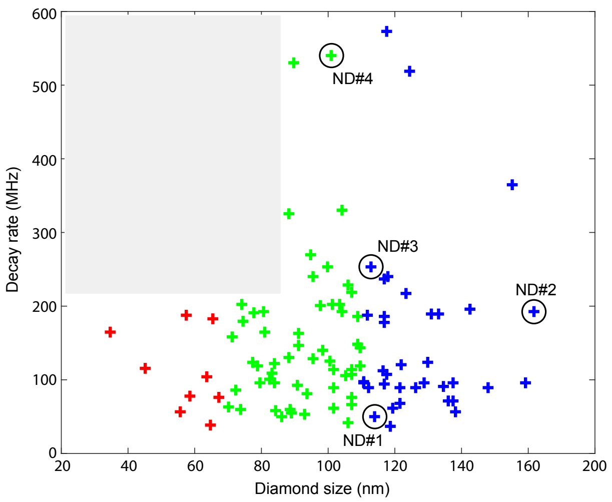

Supplementary Figure 2: **Decay rate as a function of diamond diameter** Each cross is one of the 100 diamonds in the experimental sample. NDs discussed in the main text are labeled. There is a forbidden region (shaded) showing that below a certain size, decays cannot be fast. This is consistent with our model of collective decays, and inconsistent with a model predicting that the fast decays arise from lattice defects, as such a model is independent of size. Color coding for nanodiamond diameter: red $<$ 70 nm, 70 nm $<$ green $<$ 110 nm, blue $>$ 110 nm.

Supplementary Figure 3

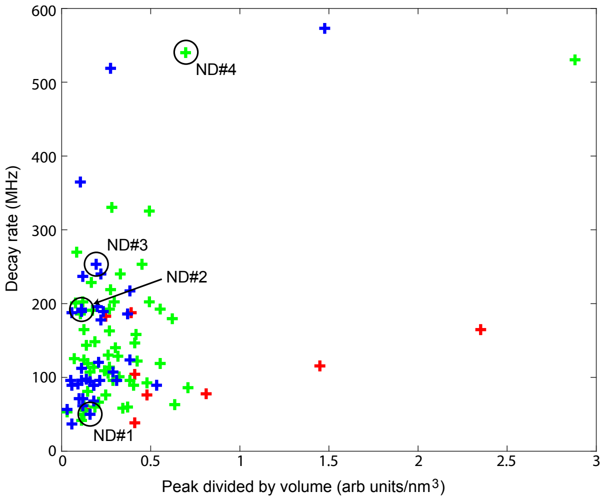

Supplementary Figure 3: **Decay rate as a function of peak brightness during the lifetime measurement** Each cross is one of the 100 diamonds in the experimental sample. NDs discussed in the main text are labeled. Brightness has been normalised to diamond volume to account for the fact that larger diamonds have more emitters and are intrinsically brighter. The fact that faster diamonds are not less bright disagrees with a lattice defect model (which predicts dark channel decay), and supports our collective decay model. Colour coding for nanodiamond diameter: red $<$ 70 nm, 70 nm $<$ green $<$ 110 nm, blue $>$ 110 nm.

Supplementary Figure 4

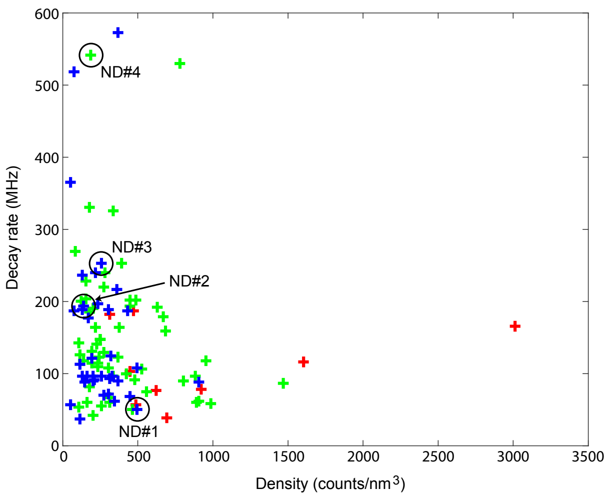

Supplementary Figure 4: **Decay rate (reciprocal lifetime) as a function of the density of NV centres in each nanodiamond** Each cross is one of the 100 diamonds in the experimental sample. NDs discussed in the main text are labeled. Densities were determined by continuous fluorescence measurements. As the quantum efficiencies of the system are unknown, the densities are in arbitrary units, but their relative values are accurate. The lack of fast diamonds at high centre densities is consistent with our model of local dephasing arising from dipole-dipole interactions destroying collective effects. Color coding for nanodiamond diameter: red $<$ 70 nm, 70 nm $<$ green $<$ 110 nm, blue $>$ 110 nm.

Supplementary Table 1. Cooperative Domains in several NDs

Diamond	N_{max}	$\gamma_{\mathrm{d}}^{0}/2\pi$	$\gamma_{\mathrm{d}}^{\pm 1}/2\pi$	$\gamma/2\pi$	p_0
NV 1	2	27 MHz	270 MHz	2.5 MHz	0.56
NV 2	7	20 MHz	260 MHz	4.8 MHz	0.51
NV 3	10	39 MHz	420 MHz	3.3 MHz	0.50
NV 4	50	20 MHz	450 MHz	7.9 MHz	0.50

Supplementary Note 1. Theoretical model

In our model, each NV centre is treated as shown in Figure 1. A green 532 nm laser excites the system into one of several vibronic levels of the excited electronic state. Within a few tens of picoseconds the system relaxes down to the vibronic ground state of the excited electronic state. From here the centre can decay electromagnetically to the various phononic states of the electronic ground state. As we are working at room temperature, we expect about 3% of the decay goes to the phononic ground state, i.e. the zero phonon line (ZPL), and 97% of the decay goes into the various excited vibronic sidebands [1].

The electronic excited state can also decay via dark, non-radiative transitions to the $^1A_1$ manifold, where it eventually relaxes to the electronic ground state over a time $\sim 100\,\mathrm{ns}$. These so-called intersystem crossing (ISC) rates, $\gamma^{\sigma}_{\mathrm{ISC}}$, depend on the spin state σ and while they are not directly observed, they can be determined from measured decay rates according to the formula [2]:

$$\frac{T_{\pm 1}}{T_0} = \frac{1+f_0}{1+f_1}, \tag{1}$$

where T_σ is the total lifetime for excited state with spin projection $m_s \equiv \sigma$, $|e_\sigma\rangle \equiv |^3E,\sigma\rangle$, and $f_\sigma = \gamma^{\sigma}_{\mathrm{ISC}}/\gamma$ where γ is the spin-independent optical decay rate [3] measured for bulk diamond to be $\gamma = 2\pi \times 12.2\,\mathrm{MHz}$ [4]. For nanodiamonds γ can be considerably slower than in bulk. This rate reduction is due the reduced size affecting the density of states as well as surface effects and other factors. In our samples we measured optical decay rates ranging from $\gamma \sim 2\pi \times 3.2$ MHz – $2\pi \times 12.7\,\mathrm{MHz}$. Since the ISC rates have not been measured in nanodiamond, we use the experimentally measured values in bulk diamond at room temperature: $\gamma^{\pm 1}_{\mathrm{ISC}} = 2\pi \times 9.4\,\mathrm{MHz}$ and $\gamma^{0}_{\mathrm{ISC}} = 2\pi \times 1.8\,\mathrm{MHz}$ [3, 6].

We coarse-grain the ensemble of NV centres in each nanodiamond into degenerate spectral domains of various sizes. Within each domain k there are N_k centres that radiate collectively after their initial excitation. We assume the optical decay from the ^3E manifold to the $^3A_2$ is collective, but the ISC decay is not. Since the optical transitions considered here are to a very good approximation spin-conserving [2], for our purposes each centre is treated as a two-level pseudospin labeled by the relevant ground and excited states for electron spin projection σ,

$$|e_\sigma\rangle \equiv |^3E,\sigma\rangle, \quad |g_\sigma\rangle \equiv |^3A,\sigma\rangle. \tag{2}$$

To describe the collective dynamics within a domain we introduce the collective spin operators

$$\hat{S}^{(\sigma)\pm} = \sum_{j=1}^{N_\sigma} \hat{s}_j^{(\sigma)\pm}, \quad \hat{S}^{(\sigma)z} = \sum_{j=1}^{N_\sigma} \hat{s}_j^{(\sigma)z}, \tag{3}$$

where for brevity the label σ is now taken to indicate both spin and spectral degeneracy. That is, each collective spin operator is a sum over local operators for the spectrally degenerate NV centres with a given spin polarisation σ,

$$\hat{s}_j^{(\sigma)+} = |e_\sigma\rangle\langle g_\sigma|_j = \left(\hat{s}_j^{(\sigma)-}\right)^\dagger, \tag{4}$$

$$\hat{s}_j^{(\sigma)z} = (|e_\sigma\rangle\langle e_\sigma|_j - |g_\sigma\rangle\langle g_\sigma|_j)/2. \tag{5}$$

Within a spectral/spin domain each degenerate NV is treated as a two-level pseudospin according to Eq. (2), and the collective state is described using Dicke states [5]. For a domain with N_σ centres the maximal total spin of a domain is given by $J = N_\sigma/2$, with $\hat{S}^{(\sigma)2} = J(J+1)$. We label the eigenstates of the collective spin operator $\hat{S}^{(\sigma)z}$ by the eigenvalues $M \in \{-J, -J+1 \ldots J-1, J\}$. The collective Dicke states $|J,M,\sigma\rangle$ can be written as

$$|J,M,\sigma\rangle = \sqrt{\frac{(J+M)!(J-M)!}{(2J)!}} \sum_{perm} |\underbrace{e_\sigma e_\sigma e_\sigma \cdots}_{J+M} \underbrace{g_\sigma g_\sigma g_\sigma \cdots}_{J-M}\rangle, \tag{6}$$

where the sum is over all permutations of the N_σ spins.

In the following we treat the $\sigma = \pm 1$ states as a single population since, at high temperature, the spin-orbit coupling in the excited state does not couple them. Additionally, their optical transitions are degenerate and thus emit spectrally indistinguishable photons, and they couple equally to the local environment [2]. This means that we can have Dicke states $|J,M,\sigma = \pm 1\rangle$ as in Eq. (6) where the permutation does not distinguish between the ± 1 spin projections. Henceforth, to simplify notation σ labels the absolute value of spin projection: $\sigma = |m_s|$ (spectral domain implied). Note that while the emitted photons from the $|E,\sigma = 1\rangle$ and $|E,\sigma = 0\rangle$ have identical polarisation, the different splittings between the $\sigma = 1$ and $\sigma = 0$ spin states in the excited versus ground manifolds ($\approx 1.46\,\mathrm{GHz}$) [2] make the bare optical transitions spectrally distinguishable. This is why the $\sigma = 0$ population is treated separately and we use the Dicke states labeled by spin projection in Eq. (6). At room temperature the broadening of the

ZPL optical transition (~ 1 THz) due to photon+acoustic phonon transitions could allow for spectrally indistinguishable photons emitted from the two spin subspaces; however, the corresponding phonons involved in such transitions could be distinguishable and hence would decohere collective Dicke states defined over both subspaces.

The initial state of our system, after excitation of the laser and after relaxation of excited vibronic states to the ^3E manifold optically excited state, is assumed to be a mixture over spin ensembles $\hat{\rho}^{(\sigma)}(0)$, each one being a mixture over Dicke states, i.e.

$$\hat{\rho}^{(\sigma)}(0) = \sum_{N_\sigma} p_{N_\sigma} \hat{\rho}^{(\sigma)}_{N_\sigma}(0), \tag{7}$$

where p_{N_σ} is the probability a domain of size N_σ exists with spin σ. Note $\sum_{N_\sigma} p_{N_\sigma} = p_\sigma$, i.e. the total fraction of contributing spins in state σ, and the overall normalisation is $\sum_{\sigma=0,1} p_\sigma = 1$. The collective state is

$$\hat{\rho}^{(\sigma)}_{N_\sigma}(0) = \sum_M P^{(\sigma)}_{J=\frac{N_\sigma}{2},M}(0) |\tfrac{N_\sigma}{2}, M, \sigma\rangle\langle \tfrac{N_\sigma}{2}, M, \sigma|, \tag{8}$$

i.e. a state diagonal in the maximum angular momentum subspace. The model was found to fit the fluorescence data by assuming a maximally mixed state in this subspace with occupation probabilities: $P^{(\sigma)}_{N_\sigma/2,M}(0) = \frac{1}{N_\sigma+1}$. The justification for this form of the initial state is as follows. Before the excitation laser is turned on, the state of the spins within a domain σ is $|\Psi\rangle_1 = |J, -J, \sigma\rangle$. The interaction of the spins with the laser is symmetric and prepares the coherent superposition $|\Psi\rangle_2 = \sum_{M,n_\nu} c^{(\sigma)}_{M,n_\nu} |J, M, \sigma, n_\nu\rangle$ where $|J, M, \sigma, n_\nu\rangle$ is a symmetric state like in Eq. (6) but where the excited state is $|e_{\sigma,n_\nu}\rangle = |^3E, n_\nu, \sigma\rangle$, where n_ν is the vibrational quantum number associated with a vibrational mode ν that the laser most strongly couples to. The coefficients $c^{(\sigma)}_{M,n_\nu}$ depend on the Rabi frequency, Franck-Condon factor, and laser detuning. In Ref. [9] it was found that the vibronic mode frequencies of the spin-triplet states of NV^- defect occur in the range $5-46$ THz and hence are well separated in energy with respect to the laser coupling. These modes are local to each spin in the sense that they are associated with deformations in the lattice within a few unit cells of the defect, which leads to a modification of the elastic moduli. The excited, local vibronic states are short-lived, with lifetimes on the order of a few picoseconds [9]. The nature of the relaxation is decay via coupling to local environmental modes which are in turn strongly coupled to long range phonon modes in the lattice. The combined effect is to erase the information about which spin experienced relaxation, and hence the decay process preserves the permutation symmetry of the collective states so that $|J, M, \sigma, n_\nu\rangle \to |J, M, \sigma\rangle$ for any initially excited vibrational state. Concurrent global dephasing processes due to non-energy-exchanging (dispersive) phonon couplings occur at room temperature a rate of ~ 1 THz [10]. This damps coherences between collective states, resulting in diagonal states of the form $\hat{\rho}^{(\sigma)}(0)$ in Eq. (8). We assume the evenly mixed state, as it fit the experimentally observed fluorescence data and the $g^{(2)}(0)$ measurements presented in the main text. Small deviations from this initial statistical distribution do not significantly change the results.

To fit the experimentally measured LT curves, we solve for the dynamics of the collective state $\hat{\rho}^{(\sigma)}$ within each spin/spectral domain undergoing collective optical decay. We then incorporate local dephasing processes in analogy to the method of Ref. [13]. We then average based on the initial polarisation of the spins. The dynamics due purely to collective optical decay within each domain are described by the superradiance master equation ($\hbar = 1$) ,

$$\frac{d\hat{\rho}^{(\sigma)}}{dt} = \mathcal{L}[\hat{\rho}^{(\sigma)}], \tag{9}$$

where the Liouvillian is

$$\mathcal{L}[\hat{\rho}^{(\sigma)}] = \frac{\gamma}{2}\left[2\hat{S}^{(\sigma)-}\hat{\rho}^{(\sigma)}\hat{S}^{(\sigma)+} - \{\hat{S}^{(\sigma)+}\hat{S}^{(\sigma)-}, \hat{\rho}^{(\sigma)}\}\right],$$

with collective operators given in Eq. (3). Since we are interested in calculating fluorescence rates we need only track populations in the collective states $P^{(\sigma)}_{J,M}(t) = \langle J, M, \sigma|\hat{\rho}^{(\sigma)}(t)|J, M, \sigma\rangle$ or single-spin excited states, and not coherences between them. Thus we need only solve rate equations and not the full master equation for the density matrix. The rate equation for collective decay only is

$$\frac{dP^{(\sigma)}_{J,M}(t)}{dt} = \gamma\left[(J(J+1) - M(M+1))P^{(\sigma)}_{J,M+1}(t) - (J(J+1) - M(M-1))P^{(\sigma)}_{J,M}(t)\right]. \tag{10}$$

Equation (10) assumes that the collective subspace remains intact throughout the entire decay process. More realistically, local decoherence processes break the symmetry of the Dicke states and remove spins from the symmetric subspace. These local decoherence processes are modelled in the following way. In the dynamical evolution we assume that all population resides either within a collective subspace spanned by the states $\{|J, M, \sigma\rangle\}$ or as independent spins. Thus, for each domain with N_σ spins, there are a series of collective subspaces with dimensions $2, 3, \ldots, 2J+1$. Each subspace has $N \leq N_\sigma$ collective

spins and total spin $J = N/2$, with $J_{\max} = N_\sigma/2$. Within the mathematical framework, we consider two local decoherence processes. The first is the aforementioned intersystem crossing at rate $\gamma^\sigma_{\rm ISC}$, which acts as local leakage to project any given spin from the excited state to the singlet state $^1A_1$ which is dark and does not contribute to the fluorescence signal. The second is a local dephasing+projection map which preserves excitation number but can couple a collective state $|J,M,\sigma\rangle$ to the state $|e_\sigma\rangle_j \otimes |J-1/2, M-1/2, \sigma\rangle$ which has one fewer spin in the collective space and one spin in the excited state. We describe this as a composite map occurring at some rate $\gamma_{\rm d}$ involving local spin dephasing followed by local projection onto the excited state. A local unitary phase flip on the collective state is $2\hat{s}_j^{(\sigma)z}|J,M\rangle$ and the overlap with the collective state is

$$\langle J,M,\sigma|2\hat{s}_j^{(\sigma)z}|J,M,\sigma\rangle = -M/J. \tag{11}$$

This means a constituent excited-state spin becomes decoupled from the state $|J,M,\sigma\rangle$ in collective J-subspace at a rate $\gamma^\sigma_{\rm d}|M/J|^2$ and joins the non-collective population where it will undergo normal exponential decay. The loss of a spin projects the remaining collective spins into the subspace of size $2J-1$ at the complementary rate $\gamma^\sigma_{\rm d}(1-|M/J|^2)$.

The rate equations in Eq. (10) for a subspace with total spin J, modified to including local decoherence are

$$\begin{aligned}\frac{d}{dt}P^{(\sigma)}_{J,M}(t) =& \gamma\Big[(J(J+1)-M(M+1))P^{(\sigma)}_{J,M+1}(t) - (J(J+1)-M(M-1))P^{(\sigma)}_{J,M}(t)\Big] \\ &- 2J\gamma^\sigma_{\rm d}\left[(1-|M/J|^2)P^{(\sigma)}_{J,M}(t) + 2(J+\tfrac{1}{2})\left(1-\left|\frac{M+\frac{1}{2}}{J+\frac{1}{2}}\right|^2\right)P^{(\sigma)}_{J+\frac{1}{2},M+\frac{1}{2}}(t)\right] \\ &+ \gamma^\sigma_{\rm ISC}\left[(J+M+1)P^{(\sigma)}_{J+\frac{1}{2},M+\frac{1}{2}}(t) - (J+M)P^{(\sigma)}_{J,M}(t)\right].\end{aligned} \tag{12}$$

In this form it is clear that the local dephasing+projection map causes feeding of populations in a given collective $J-$subspace from the that total spin one larger, $J+1$, and loss of population to the subspace with one spin smaller, $J-1$. There is also global dephasing at a rate of ~ 1 THz [10] due to coupling to long wavelength phonon modes, but because this only affects coherences between collective states within a J-subspace, this does not enter into the rate equations.

We also need keep track of the number of spins decaying independently within each domain. The relevant quantity is the number of independent spins in the excited state, which we denote $N_{\rm nc}$. This number is fed by the dephasing+projection process acting on the collective J-subspaces and is depleted by single-spin decay, includes both optical and non-radiative decay:

$$\frac{d}{dt}N^{(\sigma)}_{\rm nc} = -(\gamma+\gamma^\sigma_{\rm ISC})N^{(\sigma)}_{\rm nc} + \gamma^\sigma_{\rm d}\sum_{J=1/2}^{N/2}\sum_{M=-J}^{J}\left(1-\left|\frac{M}{J}\right|^2\right)2JP^{(\sigma)}_{J,M}. \tag{13}$$

For a given domain size $N_\sigma = 2J$ and spin σ, the fluorescence rate is

$$F_{N_\sigma}(t) = \gamma\Big(N^{(\sigma)}_{\rm nc}(t) + {\rm Tr}\big[\hat{S}^{(\sigma)+}\hat{S}^{(\sigma)-}\hat{\rho}^{(\sigma)}(t)\big]\Big) \tag{14}$$

$$= \gamma\left(N^{(\sigma)}_{\rm nc}(t) + \sum_{J=1/2}^{N/2}\sum_{M=-J}^{J}(J(J+1)-M(M+1))P^{(\sigma)}_{J,M}(t)\right). \tag{15}$$

The total fluorescence is then obtained by a weighted sum over spin ensembles and domain sizes therein,

$$F(t) = \sum_{N_\sigma} p_{N_\sigma}F_{N_\sigma}(t). \tag{16}$$

In order to solve this set of rate equations we write the populations in each spin subspace as a vector $\vec{v}^{(\sigma)}(t)$ with $({N_\sigma}^2+3N_\sigma)/2$ entries, where again N_σ is the number of spins in the domain. We then create a matrix $\mathbf{A}^{(\sigma)}$ describing the evolution of $\vec{v}^{(\sigma)}(t)$ so that

$$\frac{d}{dt}\vec{v}^{(\sigma)}(t) = \mathbf{A}^{(\sigma)}\vec{v}^{(\sigma)}(t). \tag{17}$$

As $\mathbf{A}^{(\sigma)}$ is time-independent, Eq. (17) can be solved by simple matrix exponentiation, with a computational resourse cost of ${N_\sigma}^6$ for each time point. In practice, however, scaling is considerably worse for $N_\sigma > 50$ because we begin to exceed cache space on our CPU. This scaling limits us to $\lesssim 70$ centres for a single simulation, or $\lesssim 50$ when fitting a multidimensional parameter

space. These limits are more than adequate for almost all the diamonds in our ensemble, with most showing collective effects of $N_\sigma \lesssim 10$.

Once we have this solution, the time-dependent fluorescence rate $F(t)$ is easily obtained using Eq. (15) as where $N_{\mathrm{nc}}(t)$ is the number of excited, noncollective spins which are undergoing normal exponential decay at that time. This incoherent excited population is tracked through numerical integration of the number of spins being projected out of the collective subspaces over time, along with losses due to the optical and ISC decay rates. This final theory lifetime-curve is then convolved with the experiment's detector-response function, which has been determined to be 110 ps based on a measurement with a 5 ps laser pulse.

We model the $\sigma = 0$ and $\sigma = 1$ populations separately and add their fluorescence contributions independently. We assume the ISC rates are the same as in bulk diamond. For each separate nanodiamond, the optical decay rate γ is found by fitting an exponential to the long-term tail of the lifetime fluorescence curve, when the collective effects have ended and only the non-collective optical and ISC decay channels remain.

Supplementary Note 2. Fitting the cooperative domain size

In order to fit this model to the experimentally measured lifetime curves we need to choose the number of collective centres per domain, N_σ. Rather than fit each domain separately, we assume that the domain sizes (i.e. the number of spins acting collectively in each domain) are distributed according to a probability distribution taken to be the same across all the nanodiamonds. The distribution is characterized by a single parameter N_{max}, separately fit for each nanodiamond, which corresponds to the maximum number of collective spins in the largest domain. The other possible free parameters are the degree of $\sigma = 0$ spin polarisation given by p_0 as well as the dephasing rates γ_{d}^{0} and $\gamma_{\mathrm{d}}^{\pm 1}$. When performing the fits, however, these parameters remained relatively constant across all the diamonds. Typically the degree of spin polarisation was $\sim$50% – 60%, in agreement with [16], and the dephasing rates ranged from $\gamma_{\mathrm{d}}^{0} \sim 2\pi \times 20\,\mathrm{MHz} - 2\pi \times 40\,\mathrm{MHz}$ and $\gamma_{\mathrm{d}}^{\pm 1} \sim 2\pi \times 300\,\mathrm{MHz} - 2\pi \times 450\,\mathrm{MHz}$.

We performed parameter fits for dozens of NVs centres. For the four representative diamonds we chose to profile, (Figure 2 in main manuscript), fit parameters were extracted (Supplementary Table 1). The values of maximum domain size N_{max} and polarisation p_0 found to best fit the data were determined as follows. We defined two sets S_0 and S_1 consisting of collective domain sizes for spins with projection $\sigma = 0$ or $\sigma = 1$. These sets were chosen with a variable maximum domain size and other sizes symmetrically distributed about that maximum value according to a probability distribution fixed for all the diamonds. This means the only two adjustable parameter were the maximum domain size in each set. The total number of spins in each set are $N_{\sigma\mathrm{tot}} = \sum_{n\in S_\sigma} n$ and the maximum domain size is the largest element of both the sets S_0 and S_1. Because the samples were always somewhat polarised along $\sigma = 0$, this maximum is $N_{\mathrm{max}} = \max S_0$. Given these sets of possible domain sizes, the probability for a given domain size N_σ is $p_{N_\sigma} = \frac{\sum_{n\in S_\sigma} n\delta_{n,N_\sigma}}{N_{0\mathrm{tot}}+N_{1\mathrm{tot}}}$, and the polarisation is calculated to be

$$p_0 = \frac{\sum_{n\in S_0} n}{N_{0\mathrm{tot}} + N_{1\mathrm{tot}}}. \tag{18}$$

Supplementary Note 3. Effects of dephasing

Our model for local dephasing is phenomenological. To better understand the physical mechanism, consider the effect of optical dipole-dipole interactions. For a pair of NV centres, at positions $\vec{r}_1$ and $\vec{r}_2$, the individual spin-conserving dipole-dipole interaction is (see e.g. [11])

$$\hat{H}_{\rm dd} = \sum_{\sigma_1,\sigma_2} V_{\rm dd}(|e_{\sigma_1}, g_{\sigma_2}\rangle\langle g_{\sigma_1}, e_{\sigma_2}| + |g_{\sigma_1}, e_{\sigma_2}\rangle\langle e_{\sigma_1}, g_{\sigma_2}|) \tag{19}$$

where

$$V_{\rm dd} = \frac{3\gamma b}{4(nk_0\Delta r)^3}\left[\hat{d}_1\cdot\hat{d}_2 - 3(\hat{d}_1\cdot\hat{n})(\hat{d}_2\cdot\hat{n})\right]. \tag{20}$$

Here $\hat{d}_j$ is the unit vector direction of the dipole j, $\Delta r = |\vec{r}_1 - \vec{r}_2|$ is the separation, $\hat{n} = (\vec{r}_1 - \vec{r}_2)/\Delta r$ is the unit vector separation between dipoles, $n = 2.4$ is the index of refraction of the diamond crystal, and $k_0 = 2\pi/\lambda_0$ is the vacuum wavevector of the optical transition at wavelength in nanodiamond $\lambda_0 = 639$ nm. For the present estimate we use the bulk lifetime $\tau = 12.9$ ns to find a total optical decay rate $\gamma = 1/\tau = 2\pi \times 12.3$ MHz. With the branching ratio into the ZPL of $b \approx 0.03$ [1] we find an interaction strength of

$$V_{\rm dd} = 2\pi \times 20.9\ \text{MHz} \times \left(\frac{10\text{nm}}{\Delta r}\right)^3 \left[\hat{d}_1\cdot\hat{d}_2 - 3(\hat{d}_1\cdot\hat{n})(\hat{d}_2\cdot\hat{n})\right]. \tag{21}$$

From the manufacturer provided numbers, the density of NV centres in the nanodiamonds is $\rho_{\rm NV} \sim 10^{24}$ m^{-3}, which gives a mean separation between any pair of 10 nm. Parallel dipoles at this separation experience an interaction strength of $V_{\rm dd} \approx 2\pi \times 20$ MHz.

The dipole-dipole interaction, Eq. (19), preserves the number of excitations M but couples collective states within different J-subspaces, and in general is not permutation symmetric for randomly located NV centres, even when they are members of a degenerate domain. Although our model does not capture these features, we emphasise that the strength of the expected dipole-dipole interaction, Eq. (21) is at the lower end of our inferred local dephasing rates $\gamma_{\rm d}^0$, even after summing over dipole pairs at different separations and angles for our domain sizes. This implies that we are at least not underestimating the effects of dipole-dipole interactions, as the inferred dephasing rates $\gamma_{\rm d}^{\pm 1}$ are an order of magnitude larger. This is likely due to coupling to local electric fields at each spin, which has a larger coupling strength due to the significant excited state permanent dipole moment for the ± 1 states [2].

To compare our model to non-collective behaviour, we attempted to fit the observed lifetimes with both a bi-exponential and a deformed exponential lifetime curve [12]. The fits using the bi-exponential model were consistently worse than our model with the discrepancy increasing as the collectivity increased. For the deformed exponential we used a coefficient appropriate to dipole-dipole coupling and a dimensionless coupling strength parameter [12] consistent with the defect density of our samples. This also resulted in poorer fits than our model with ratios of least squares errors up to 40 times worse.

Supplementary Note 4. Second-order coherence

The normalised second-order coherence function for delay time t and for a ensemble with domain size N_σ is

$$g^{(2)}_{N_\sigma}(t) = \frac{\langle \hat{S}^{(\sigma)+}(0)\hat{S}^{(\sigma)+}(t)\hat{S}^{(\sigma)-}(t)\hat{S}^{(\sigma)-}(0)\rangle}{\langle \hat{S}^{(\sigma)+}(0)\hat{S}^{(\sigma)-}(0)\rangle\langle \hat{S}^{(\sigma)+}(t)\hat{S}^{(\sigma)-}(t)\rangle}. \tag{22}$$

The total observed second-order coherence is the sum

$$g^{(2)}(t) = \sum_\sigma \sum_{N_\sigma} p_{N_\sigma} g^{(2)}_{N_\sigma}(t). \tag{23}$$

Assuming the population is initially in the largest collective J-subspace, then immediately after the excitation process is complete and before decay begins we have (here $J = N_\sigma/2$)

$$\mathrm{Tr}\left[\hat{S}^{(\sigma)+}\hat{S}^{(\sigma)+}\hat{S}^{(\sigma)-}\hat{S}^{(\sigma)-}\hat{\rho}^{(\sigma)}_{N_\sigma}(0)\right] = \sum_{M=-J}^{J} P^{(\sigma)}_{J,M}(0)\left[J(J+1) - M(M-1)\right] \times \left[J(J+1) - (M-1)(M-2)\right], \tag{24}$$

$$\mathrm{Tr}\left[\hat{S}^{(\sigma)+}\hat{S}^{(\sigma)-}\hat{\rho}^{(\sigma)}_{N_\sigma}(0)\right] = \sum_{M=-J}^{J} P^{(\sigma)}_{J,M}(0)\left[J(J+1) - M(M-1)\right]. \tag{25}$$

For zero delay, the result for $g^{(2)}(0)$ is clearly dependent on the choice of initial state. In the extreme case where the initial state consists of all spins up (corresponding to complete inversion in the collective subspace), we have $P^{(\sigma)}_{J,M} = \delta_{J,M}$, so that

$$g^{(2)}_{N_\sigma}(0) = 2 - \frac{2}{N_\sigma}. \tag{26}$$

Thus, for large domain sizes, the second-order coherence function approaches 2.

As described above, however, in our modelling we assume an initial state where all M eigenstates are equally populated in the maximum J-subspace. That is,

$$P^{(\sigma)}_{J,M}(0) = \frac{\delta_{J,N_\sigma/2}}{2J+1}\ \forall M \in \{-J,\ldots,J\}. \tag{27}$$

With this initial state we find

$$g^{(2)}_{N_\sigma}(0) = \frac{6(N_\sigma - 1)(N_\sigma + 3)}{5N_\sigma(N_\sigma + 2)}, \tag{28}$$

which approaches $g^{(2)}_{N_\sigma}(0) = 1.2$ as $N_\sigma \to \infty$. For a set of varying domain sizes N_σ with probability distribution p_{N_σ}, and initial states given by Eq. (8), the second order coherence is

$$g^{(2)}(0) = \sum_\sigma \sum_{N_\sigma} p_{N_\sigma} \frac{6(N_\sigma - 1)(N_\sigma + 3)}{5N_\sigma(N_\sigma + 2)}. \tag{29}$$

The solid red line in Fig. 3e of the main text plots $g^{(2)}(0)$ from Eq. (29) for the initial state in Eq. (7).

Supplementary Note 5. NV ensemble statistics

As noted in the main manuscript, previous studies reported a decrease in the lifetimes of NVs for centres produced via low-energy He-ion radiation, with the decay time decreasing for increasing ion doses. This effect has been attributed to increased damage in the crystal lattice which provided nonradiative decay paths with faster dynamics [14, 15], suggesting that the shortening of the lifetimes was due to nonradiative, 'dark' pathways. In order to demonstrate that is not the relevant decay mechanism in our experiment, and that the lifetime shortening in our system is due to coherent collective effects, we took the entire ensemble of 100 nanodiamonds and examined the relationship between decay rates and size, brightness and NV centre density.

Supplementary Figure 2 shows the relation between nanodiamond size and the decay rate, with each point on the plot corresponding to a single diamond in the ensemble. In order to not rely on a specific model for the analysis of the decay curves, we extracted the initial slope of the curve by fitting an exponential decay of the form $\exp[-t/\tau]$ to the first three nanoseconds of the lifetime curve, and then defining the rate as $1/\tau$. The lifetime curves do not follow standard exponential decay, especially for those with strong collective effects, but this method does produce a rate that reflects relative decay rates at short times.

The shaded region in the top left of Supplementary Figure 2 contains no experimental data points indicating that small nanodiamonds ($<$70 nm in diameter) cannot be fast. This is clearly inconsistent with hypothesis of crystal damage to lattice causing the fast decays. If it were true, then one would expect the decay speed up to be insensitive to diamond size — the dark channels would be present for small diamonds as well as large diamonds. The lack of fast diamonds at small sizes is consistent with surface effects. As diamonds get smaller, the surface to volume ratio increases, and surface effects can break distinguishability, reducing the size of the collective domains. Thus Supplementary Figure 2 is consistent with superradiant decay due to collective effect.

This is also confirmed by Supplementary Figure 3, which shows decay rate versus the peak fluorescence of the lifetime curve normalized to diamond volume (accounting for the fact that larger diamonds are intrisically brighter). The crystal damage hypothesis would suggest that faster diamonds should be less bright, since the acceleration of decay rate is due to dark channels. Again, no such effect is seen. If anything, a trend towards brighter diamonds being faster is seen, which is consistent with our model of collectively enhanced decay through a bright channel.

Finally we plot the decay rates versus NV centre density in Supplementary Figure 4. The data suggests a trend that beyond a certain NV centre density, no rapid decay occurs. This is consistent with the idea that a high centre density leads to larger local dephasing due to dipole-dipole interactions.

Supplementary References

[1] C. Santori C, P. Barclay P, K.C. Fu, R. Beausoleil, S. Spillane, and M. Fisch, Nanotechnology **21**, 274008 (2010).
[2] Marcus W. Doherty, Neil B. Manson, Paul Delaney, Fedor Jelezkod, Jörg Wrachtrupe, and Lloyd C.L. Hollenberg, Physics Reports **528**, 1–45 (2013).
[3] A. Batalov, C. Zierl, T. Gaebel, P. Neumann, I.-Y. Chan, G. Balasubramanian, P.R. Hemmer, F. Jelezko, and J. Wrachtrup, Phys. Rev. Lett. **100** 077401 (2008).
[4] N.B. Manson, J.P. Harrison, and M.J. Sellars, Phys. Rev. B **74** 104303 (2006).
[5] Dicke, R. H., Phys. Rev. **93**, 1 (1954).
[6] M.L. Goldman, A. Sipahigil, M.W. Doherty, N.Y. Yao, S.D. Bennett, M. Markham, D.J. Twitchen, N.B. Manson, A. Kubanek, and M.D. Lukin, Phys. Rev. Lett. **114** 145502 (2015).
[7] B. M. Garraway, Phil. Trans. R. Soc. A **369**, 1137–1155 (2011).
[8] Ph. Tamarat, *et al.* Phys. Rev. Lett. **97**, 083002 (2006).
[9] V.M. Huxter, T.A.A. Oliver, D. Budker, and G.R. Fleming, Nat. Phys. **9**, 744 (2013).
[10] K.-M.C. Fu *et al.* Phys. Rev. Lett. **103** 256404 (2009).
[11] A. Albrecht, A. Retzker, F. Jelezko, and M.B. Plenio, New J. Phys. **15** 083014 (2013).
[12] D. Gatto Monticone *et al.*, Phys. Rev. B **88**, 155201 (2013).
[13] B. Q. Baragiola, B. A. Chase, and JM Geremia, Phys. Rev. A **81**, 032104 (2010).
[14] D. McCloskey, D. Fox, N. O'Hara, V. Usov, D. Scanlan, N. McEvoy, G.S. Duesberg, G.L.W. Corss, H.Z. Zhang, and J.F. Donegan, Applied Physics Letters **104**, 031109 (2014).
[15] J.O. Orwa, K.W. Nugent, D.N. Jamieson, and S. Prawer, Phys. Rev. B **62** 5461 (2000).
[16] S. Felton, A.M. Edmonds, M.E. Newton. P.M. Martineau, D. Fisher, D.J. Twitchen, and J.M. Baker, Phys. Rev. B **79**, 075203 (2009).
